# Functional expression of a Mo-dependent formate dehydrogenase in *Escherichia coli* under aerobic conditions

**DOI:** 10.1371/journal.pone.0334613

**Published:** 2025-10-29

**Authors:** Marion Schulz, Anne Berger, David Roche, Emilie Pateau, Ivan Dubois, Valérie A. Delmas, Mélodie Cadillon, Madeleine Bouzon, Volker Döring

**Affiliations:** Génomique Métabolique, Genoscope, Institut François Jacob, CEA, CNRS, Univ Evry, Université Paris-Saclay, Evry-Courcouronnes, France; Federal University Dutse, NIGERIA

## Abstract

**Background:**

Oxygen tolerant complex metal-dependent formate dehydrogenases hold potential for biotechnological applications.

**Principal Findings:**

In this work, we report the functional expression of the complex, molybdenum-dependent soluble formate dehydrogenase encoded by the *fdsGBACD* operon from *Cupriavidus necator* (CnFDH) in *Escherichia coli.* Expression of the operon from plasmids or from a copy integrated in the chromosome enabled growth of an energy-auxotrophic selection strain on formate as sole energy source under aerobic conditions. Growth could be accelerated in turbidostat, leading to a drop of the generation time of 1 hour. While no mutation was found in the operon of evolved isolates, genome sequencing revealed non-synonymous point mutations in the gene *focA* coding for a bidirectional formate transporter carried in all isolates sequenced. Reverting the mutations led to a drop in the growth rate demonstrating the *focA* gene as principal target of continuous culture adaptation.

**Significance:**

A member of the oxygen-tolerant subclass of complex FDH showed stable formate oxidation activity when expressed in the heterologous host *E. coli*, a model organism of biotechnology. The integration of the operon in the chromosome offers the possibility of structure/function studies and activity enhancements through *in vivo* mutagenesis, which can also be applied to CO_2_ reduction in appropriate selection hosts.

## Introduction

Formate dehydrogenases (FDH), a diverse family of enzymes, catalyze the reversible conversion of formate to CO_2_, using the nicotinamide cofactors NAD(P)/NAD(P)H or other compounds as redox co-substrate [1]. Members of this family can be divided into metal-dependent and metal-independent FDHs. The latter are monomeric proteins that do not contain redox-active centers, they are oxygen-insensitive and depend on NADH as redox cofactor [2]. Due to their simple structures and complete O_2_ tolerance, metal-independent enzymes are the ones mostly used in biotechnological applications, notably for the regeneration of NADH, but also in the reductive direction in electrochemical [3] and photo-electrochemical processes [4]. By contrast, the metal-dependent enzymes contain either a molybdenum or a tungsten atom as part of a pyranopterin guanosine dinucleotide (PGD) cofactor, at least one Fe/S-center and have a complex quaternary structure [5]. Although most of these enzymes are oxygen sensitive, membrane bound and require electron donors/acceptors other than NAD(H), a few O_2_-tolerant, NAD(H)-dependent and soluble enzymes have been found among this class, notably from the metabolically versatile bacteria *Cupriavidus necator* N1 (CnFDH) [6–8] and *Rhodobacter capsulatus* (RcFDH). The cryo-EM structure of this latter enzyme was solved, providing first insights into their mechanism of catalysis [9].

Most FDHs preferentially catalyze the exergonic oxidation of formate to CO_2_. However, under appropriate thermodynamic conditions, they can reduce CO_2_ to formate [10], thus having the potential to become valuable catalysts in the circular carbon economy: the greenhouse gas CO_2_ is converted to value-added formate, that can be used as hydrogen storage material, as fuel in “Direct Formic Acid Fuel Cells” [11], as a versatile C_1_ synthon for chemical synthesis and as sustainable feedstock for the bioindustry [12,13]. The different FDH enzyme classes vary in this capacity [14], with the O_2_-sensitive metal-dependent dehydrogenases from anaerobic bacteria like *Acetobacter woodii* [15] being the most active (*k*_cat_ > 500 sec^-1^), and the metal-independent dehydrogenases being the least active catalysts (*k*_cat_ < 1 sec^-1^). While examples exist in the literature in which the activity of a metal-independent FDH was enhanced up to 3-fold by site directed mutagenesis [16], it can be assumed that the subclass of O_2_-tolerant, NAD- and metal-dependent enzymes have a higher potential to become the enzyme workhorses of CO_2_ reduction under aerobic conditions. The cytoplasmic FDH purified from *Cupriavidus necator* was shown to catalyze this reaction with a *k*_*cat*_ = 11 sec^-1^ under anaerobic conditions [7]. However, enzyme purification was conducted under fully aerobic conditions demonstrating oxygen tolerance despite the presence of a molybdenum-containing CO_2_-formate redox active site and four [4Fe-4S] centers and one [2Fe-2S] center in the α-subunit (FdsA, 105 kDa), a FMN cofactor for NAD/NADH electron transfer and a [4Fe-4S] center in the β-subunit (FdsB, 55 kDa) and a [2Fe-2S] center in the γ-subunit (FdsG, 19 kDa).

While *ex vivo* structural and activity studies with complex FDHs have been conducted in recent years, studies of their activity in a cellular context are scarce. Recently, a Mo-dependent enzyme homologously expressed in *Pseudomonas putida* was shown to be active in a selective, formate-dependent context [17]. *E. coli* harbors three complex membrane associated formate dehydrogenases. They are functional under anaerobic growth conditions [18] and deliver electrons to a quinone acceptor (FDH-O and FDH-N), or for H_2_ production (FDH-H), but not for NAD-reduction. Therefore, formate does not function as source of reducing power in *E. coli*. In this report, we describe the cloning and heterologous plasmid-borne expression of the *fdsGBACD* operon coding for CnFDH in an *E. coli* MG1655 derived energy auxotrophic selection strain. We obtained expression-dependent aerobic growth on formate as sole source of energy and isolated faster growing strain descendants upon evolution in continuous culture harboring genetic background mutations. Sustained formate dependent growth was also obtained when the operon was inserted into the chromosome of an evolved isolate cured from the plasmid. Expression from one copy upon genomic integration stabilizes the construct and will enable long-term strain adaptation and evolution in chosen genetic backgrounds to ameliorate enzyme activity and tolerance to O_2_.

## Results

### Rescue of an energy auxotrophic *E. coli* strain through formate oxidation by FDH from C. necator

**Fig 1:**
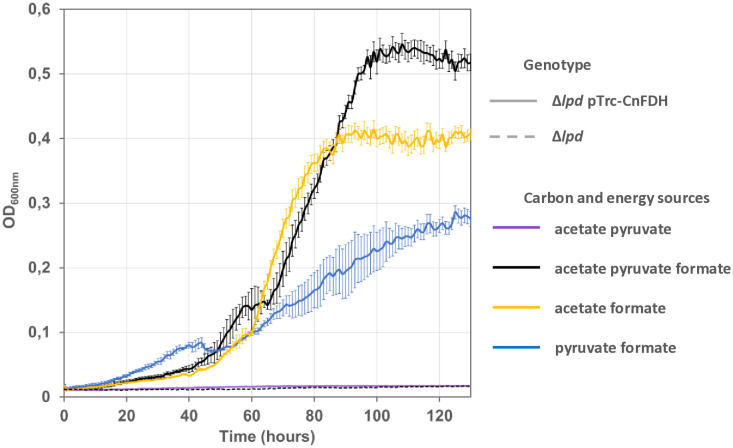
Expression of *C. necator* NAD-dependent formate dehydrogenase allows *E. coli* NADH auxotroph strain Δ*lpd* to use formate as energy source. Strains G5416 (Δ*lpd*) (broken line) and G5663 (Δ*lpd* pTRC-CnFDH) (plain line) were grown at 30°C on mineral MS medium supplemented with the indicated compounds. Concentrations of formate, acetate and pyruvate were 60, 20 and 20 mM, respectively. Growth was recorded with a Bioscreen C plate reader, shown are the mean values of three measurements and the standard deviation.

The soluble NAD- and Mo-dependent native formate dehydrogenase from *C. necator* is coded by the *fdsGBACD* operon, with the genes *fdsGBA* specifying the three enzyme subunits and the genes *fdsCD* specifying two chaperones shown to be essential for enzyme activity [19,20]. FdsD was recently shown to be part of the FdsGBAD heterotetrameric functional unit of the closely related FDH from *Rhodobacter capsulatus* [9]. The operon was amplified by PCR from chromosomal *C. necator* DNA and cloned into plasmid pTrc99a. We chose this vector for its strong inducible *trc* promoter assuring high operon expression. The resulting plasmid pTRC-CnFDH (pGEN1340) was introduced into an *E. coli* MG1655 strain deleted for the gene *lpd* coding for lipoamide dehydrogenase (strain G5416) yielding strain G5663 (for strain and plasmid description, refer to Table 1). This enzyme, a component of the pyruvate dehydrogenase and the 2-oxoglutarate dehydrogenase complexes, catalyzes electron transfer from the dihydrolipoamide carrier to NAD^+^, respectively. *E. coli* strains lacking lipoamide dehydrogenase activity require, when fed with acetate as sole carbon source, an energy source in addition for growth. In this context, NAD-dependent formate oxidation to CO_2_ can provide the necessary energy, as was shown for the monomeric NAD-dependent FDH of *Pseudomonas* sp.101 [21]. When the expression of the plasmid-borne *C. necator fdsGBACD* operon was induced by IPTG addition in the culture of strain G5663 [Δ*lpd* pTRC-CnFDH], growth was obtained in mineral medium in the presence of formate (60 mM), acetate (20 mM) and pyruvate (20 mM) (Fig 1). In contrast, no growth was observed when formate was omitted in the culture medium or in the case the energy auxotroph did not harbor the plasmid pTRC-CnFDH (Fig 1), demonstrating that the *C. necator* NAD-dependent cytoplasmic formate dehydrogenase is functional when expressed in the *E. coli* host. As previously reported [21], we observed low growth yield on formate (60 mM) and acetate (20 mM) as sole carbon source, which was enhanced through pyruvate addition. Pyruvate when replacing acetate supported sustainable growth on formate as energy source. We speculated that the production of acetate through the action of pyruvate oxidase, catalyzing the oxidative decarboxylation of pyruvate to acetate [22,23] was responsible for this supporting effect. However, deletion of the gene *poxB* specifying the enzyme did only slightly affect growth, pyruvate still being a growth-enhancing factor (not shown). Acetate might be produced from pyruvate by an activity other than pyruvate oxidase, pyruvate formate lyase (Pfl) being a candidate, even so this enzyme is described inactive in the presence of oxygen. In addition, pyruvate might function as supplementary carbon source through gluconeogenesis and as precursor of several amino acids, while it cannot function as electron donor for growth.

**Table 1:** Strains and plasmids used in this study.

Strain	Genotype	Origin/modification
MG1655	F-, LAM-, *rph-1*	CGSC Collection, Yale
JW0889	Δ*ycaP::kan*^*R*^	Keio Collection,
JW0112	Δ*lpd::kan*^*R*^	Keio Collection
G2129	Δ*lpd::kan*^*R*^	Transduction MG1655 x P1 (Keio JW0112)
G5416	Δ*lpd*	Excision of the kanamycin resistance cassette of G2129
G5663	Δ*lpd* pGEN1340 (*fdsGBACD C.n).*	Transformation of G5416
G5823	Δ*lpd* pGEN1340 (*fdsGBACD C.n)..* *focA* F7C *infC* E95K	Evolvant of G5663 selected in GM3 under turbidostat regime – UOF1 lineage
G5824	Δ*lpd* pGEN1340 (*fdsGBACD C.n).* *focA* F7C *infC* E95K	Evolvant of G5663 selected in GM3 under turbidostat regime – UOF1 lineage
G5825	Δ*lpd* pGEN1340 (*fdsGBACD C.n).* *focA* F7C *infC* E95K	Evolvant of G5663 selected in GM3 under turbidostat regime – UOF1 lineage
G5848	Δ*lpd* pGEN1340 (*fdsGBACD C.n)..* *focA* V97I *pps* S2F *crr* G2V *fabR* L53W	Evolvant of G5663 selected in GM3 under turbidostat regime – UOF2 lineage
G5849	Δ*lpd* pGEN1340 (*fdsGBACD C.n)..* *focA* V97I *pps* S2F *crr* G2V *fabR* L53W	Evolvant of G5663 selected in GM3 under turbidostat regime – UOF2 lineage
G5850	Δ*lpd* pGEN1340 (*fdsGBACD C.n)..* *focA* F7C *cra* Y28C	Evolvant of G5663 selected in GM3 under turbidostat regime – UOF2 lineage
G5873	Δ*lpd* pGEN1340 (*fdsGBACD C.n)..* *focA* V97I *pps* S2F *crr* G2V *fabR* L53W	Curated G5849
G5876	Δ*lpd focA* F7C *infC* E95K	Curated G5823
G6234	Δ*lpd* pGEN1340 (*fdsGBACD C.n)..* *infC* E95K Δ*ycaP::kan*^*R*^	Transduction G5823 x P1 (Keio JW0889)
G6235	Δ*lpd* pGEN1340 (*fdsGBACD C.n). pps* S2F *crr* G2V *fabR* L53W Δ*ycaP::kan*^*R*^	Transduction G5848 x P1 (Keio JW0889)
G6114	Δ*lpd* pGEN1393 (*fdsGBACD C.n)..*	Transformation of G5416
G6217	Δ*lpd* pGEN1395 (*fdh T.sp).*	Transformation of G5416
G6272	Δ*lpd.focA* F7C *infC* E95K pGEN1395 (*fdh T.sp)*	Transformation of G5876
G6280	Δ*lpd focA* F7C *infC* E95K pGEN1393 (*fdsGBACD C.n)*	Transformation of G5876
G6408	IS10::*fdsGBACD C.n.::kan*	Insertion at IS10 by recombination with conjugative plasmid pKI_IS10_CnFDH in MG1655
G6435	Δ*lpd focA* F7C *infC* E95K IS10::*fdsGBACD C.n.::kan*	Transduction G5876 x P1(G6408)
G6501	Δ*lpd* Δ*ycaP::cat*	Recombination of fragment Δ*ycaP::cat* in G5873
G6502	Δ*lpd* Δ*ycaP::cat*	Recombination of fragment Δ*ycaP::cat* in G5876
G6504	Δ*lpd focA* F7C *infC* E95K pGEN1340 (*fdsGBACD C.n).*	Transformation of G5876
G6514	Δ*lpd focA* V97I Δ*ycaP::cat*	Transduction G5416 x P1 G6501
G6515	Δ*lpd focA F7C* Δ*ycaP::cat*	Transduction G5416 x P1 G6502
G6759	Δ*lpd infC* E95K Δ*ycaP::kan*^*R*^	Curated G6234
G6760	Δ*lpd pps* S2F *crr* G2V *fabR* L53W Δ*ycaP::kan*^*R*^	Curated G6235
**Plasmid**	**Description**	**Origin**
pTrc99a	IPTG-inducible expression vector, pBR322 origin, *bla*^ +^* lacI*^+^	CGSC Collection, Yale
pFDH	pZE21-derived plasmid, constitutive moderate promoter, pBR322 origin, streptomycin^R^, *fdh Pseudomonas sp101,* backbone designated as pZE in this study	Addgene (#131706)
pFD152	dCas9 (aTc-inducible), spectinomycin^R^, gRNA cloning site	[38]
pKI_IS10	R6K origin, conjugative, chloramphenicol^R^ kanamycin^R^,	[36]
pGEN1340	pTrc99a::*fdsGBACD Cupriavidus necator* native operon	This study
pGEN1378	pKI_IS10::*fdsGBACD Cupriavidus necator* native operon	This study
pGEN1393	pZE::*fdsGBACD Cupriavidus necator* native operon	This study
pGEN1395	pZE::*fdhThiobacillus sp.* synthetic gene	This study

### Acceleration of formate dependent growth in continuous culture

**Fig 2:**
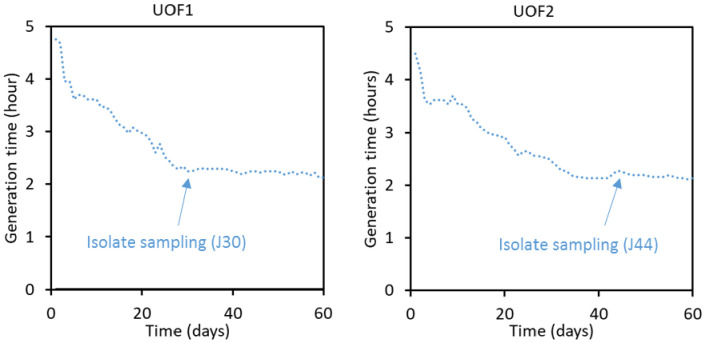
Growth acceleration of NADH auxotroph G5663 bacteria (Δ*lpd* pTRC-CnFDH) in turbidostat. Cells were grown in two independent cultures (UOF1 and UOF2) at 30°C in mineral MS medium supplemented with acetate (20 mM), pyruvate (20 mM) and formate (60 mM) in a GM3 device for 60 days. The time points of isolate samplings are indicated, corresponding to 230 generations in turbidostat for UOF1 and 380 generations in turbidostat for UOF2.

**Fig 3:**
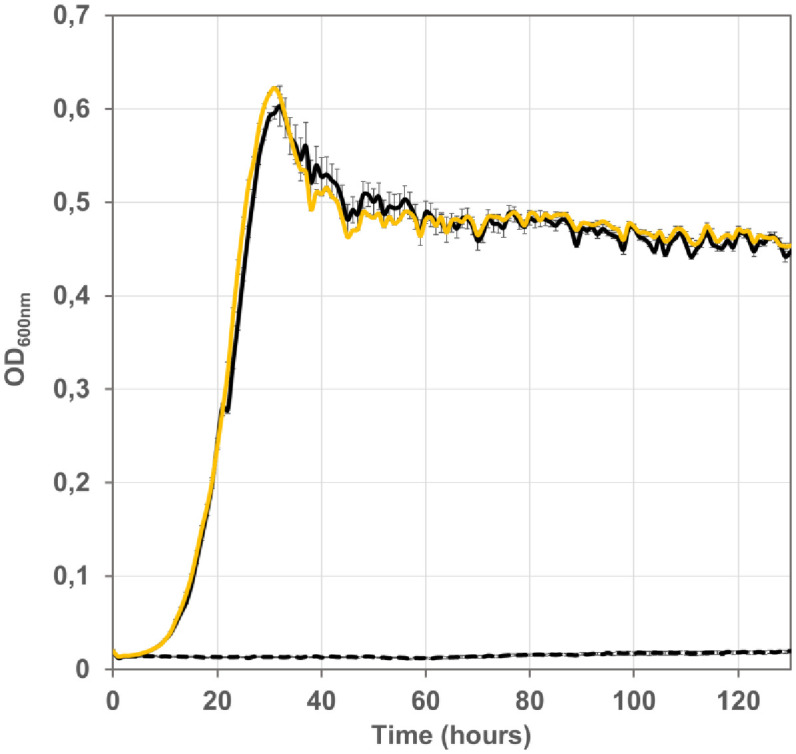
Dependence of evolved isolate G5823 (turbidostat culture UOF1) on the presence of plasmid-borne *C. necator* FDH for growth on formate as energy source. Strains G5823 (black line), G5876 (derivative of G5823 cured from the plasmid pTRC-CnFDH) (black broken line), and strain G6504 (derivative of G5876 transformed with plasmid pTRC-CnFDH) (yellow line) were grown at 30°C on mineral MS medium supplemented with formate (60 mM), acetate (20 mM) and pyruvate (20 mM). Growth was recorded with a Bioscreen C plate reader, shown are the mean values of three measurements and the standard deviation.

To accelerate formate-dependent growth, a cell population of NADH-requiring strain G5663 growing in selective medium (formate/acetate/pyruvate) in the presence of IPTG was subjected to a turbidostat in GM3 continuous culture automatons (see materials and methods). Two independent cultures (UOF1 and UOF2) were launched in parallel. Both cultures were characterized by a short adaptation phase during which the initial generation time dropped rapidly from around 4h40 to stabilize at 3h40. Growth of both cell populations continuously accelerated (Fig 2) until reaching a plateau with a generation time of 2h15 for both cultures, representing a diminution of about 1h25 as counted from the first stabilized plateau. Three isolates were obtained from each culture and formate dependence of growth verified. Isolate G5823 from UOF1 culture was cured from plasmid pTRC-CnFDH upon serial culture in selective medium supplemented with glucose. Plasmid loss was verified by sensitivity to ampicillin and the absence of PCR amplification of the *fdsGBACD* operon. The cured cells (strain G5876) lost their capacity to use formate as an energy source. Introducing plasmid pTRC-CnFDH in the cured cells restored growth on selective medium, showing that the dependency on FDH-catalyzed formate oxidation for energy and reducing power supply was maintained during strain evolution (Fig 3).

**Fig 4:**
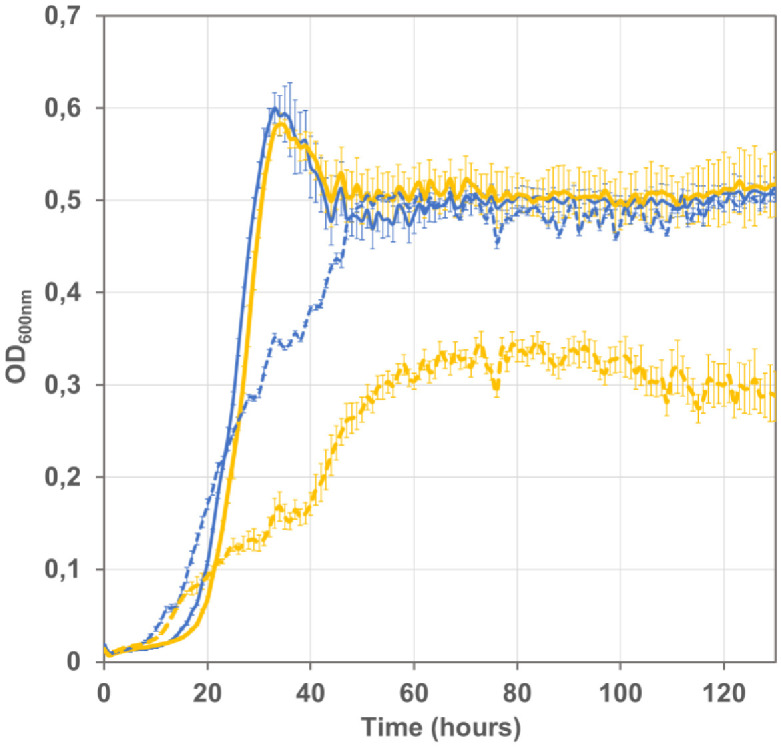
FocA wild type derivatives of evolved UOF strains show diminished growth on formate as energy source. Evolved Δ*lpd* strains G5823 (*focA* F7C, plain blue line) and G5848 (*focA* V97I, plain yellow line) and their *focA* wild type derivatives G6234 (broken blue line) and G6235 (broken yellow line), respectively, were grown in mineral MS medium supplemented with 20mM acetate 20 mM pyruvate 60 mM formate in a Bioscreen C plate reader in triplicate, shown are the mean values of three measurements and the standard deviation.

To identify adaptive mutations entailing improved growth under selective conditions, we proceeded to whole genome Illumina sequencing of all six isolates and mapped the sequence reads onto the genome (chromosome and plasmid pTRC-CnFDH) of the ancestor strain (see materials and methods). The plasmid pTRC-CnFDH from all six isolates remained unchanged. Sequencing of the genomes of the isolates identified a total of nine point mutations, with eight genes harboring a non-synonymous mutation in their coding region and one mutation affecting an intergenic region (Supplementary information, S1 file). The only gene found to be affected in all isolates, albeit not carrying the same mutation, was *focA* coding for a bidirectional formate transporter, differing in the changed codon between isolates. Interestingly, one mutation (*focA* F7C) was fixed in isolates obtained from both evolved populations from UOF1 and UOF2 independent cultures. The pH-dependent channel FocA plays an important role in the regulation of intracellular formate concentration, notably during mixed-acid fermentation [24,25]. We tested the impact of *focA* mutations F7C (isolate G5823) and V97I (isolate G5848) on formate-dependent growth by exchanging the mutated with the wild type *focA* allele in the two isolates. Growth diminished significantly for both derived strains harboring wild type *focA* when grown in the formate/acetate/pyruvate test medium (Fig 4).

**Fig 5:**
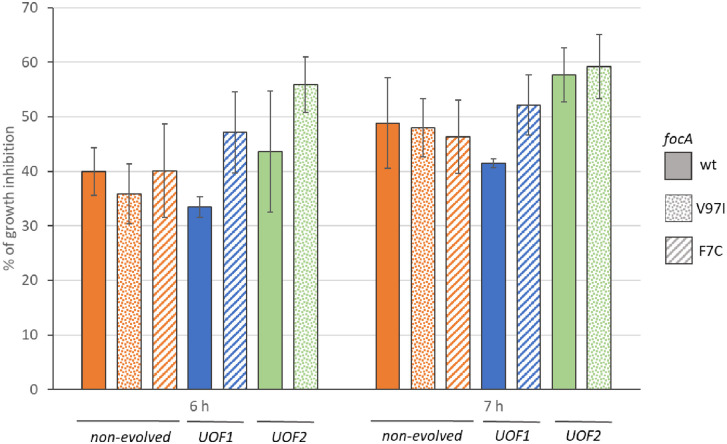
Hypophosphite toxicity for evolved and non-evolved energy auxotrophic selection strains. Non-evolved Δ*lpd* control strain G5416 and its *focA* mutated derivatives G6514 (*focA* V97I) and G6515 (*focA* F7C), the evolved derivatives G5876 (culture UOF1) and G5873 (culture UOF2) and their *focA* wild type descendants G6759 and G6760, respectively were grown in anaerobic conditions on mineral glucose medium with or without 10 mM hypophosphite. After the indicated hours of fermentative growth, OD_600nm_ was determined and hypophosphite-induced growth inhibition calculated. Shown are the mean values of three independent experiments and the standard deviation.

**Fig 6:**
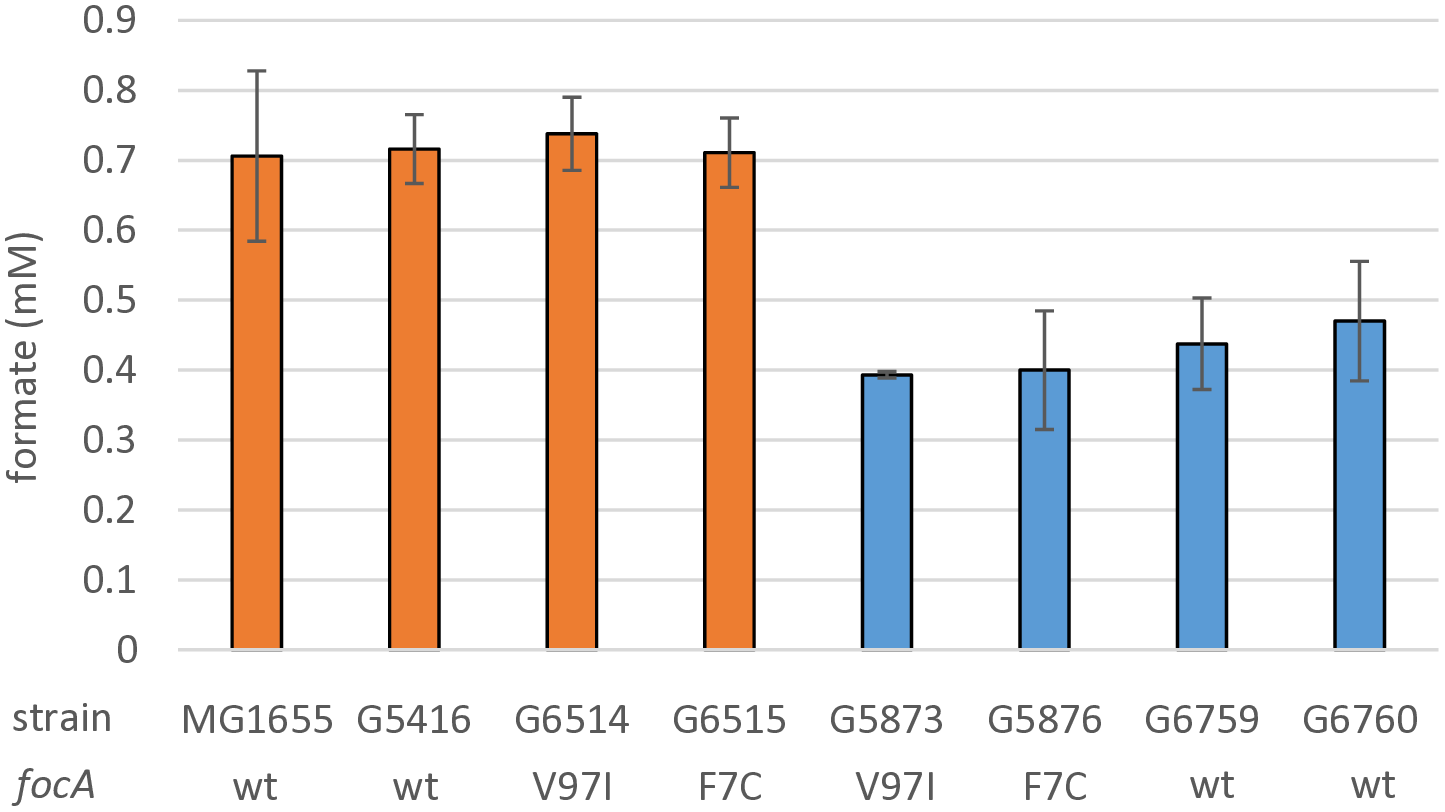
Formate secretion during anaerobic growth on glucose is lower for evolved UOF strain derivatives. Control strain MG1655, its non-evolved Δ*lpd* derivatives expressing wild type or mutant gene *focA* (orange bars) and UOF-derived Δ*lpd* strains harboring wild type or mutant gene *focA* (blue bars) were grown in anaerobic conditions on mineral glucose medium. After 8 hours of fermentative growth, formate concentrations in the growth media were measured by an enzymatic assay and the value normalized for cell density. Shown are the mean values of three independent experiments and the standard deviation.

FocA regulates formate concentration in the cytoplasm by favoring formate influx or efflux depending on medium pH and the growth phase of cultures in anaerobic environments. In *E. coli*, glucose fermentation in the absence of an electron acceptor generates formate among other acids [26]. Formate is produced from pyruvate cleavage catalyzed by pyruvate formate lyase (PflB), a radical glycine enzyme inactive in the presence of oxygen [27]. It has been found that PflB and FocA proteins interact, modulating the FocA channel activity [28]. In this line, it was shown that the genes *focA* and *pflB*, associated to the formate regulon in *E. coli* [29], are co-expressed. While *pflB* transcription occurs in aerobic conditions from an independent promoter, only very low FocA expression was documented for growth in the presence of oxygen [30]. Therefore, a possible functional presence of the FocA channel during UOF culture evolution under strict aerobic conditions needs to be rationalized. Transcription of the formate regulon is complex, involving general regulators like FhlA which in turn are synthesized upon formate accumulation in the cells [31]. We hypothesize that transcription of at least some of the genes of the regulon in aerobically growing cells in the presence of high formate (60 mM during evolution) takes place somehow analogous to the situation of anaerobic growth when formate accumulates from pyruvate cleavage, thus leading to FocA expression. In the UOF turbidostats, the population is constantly growing in the logarithmic phase (OD = 0.4) at a pH favoring FocA efflux activity (culture medium pH = 7.2). Possibly, the mutant variants which arose during evolution impact the fine-tuned regulation of the channel favoring influx or impeding efflux of formate as response to the pressure imposed by the selection. To obtain some experimental insights about the effect of the mutations, we conducted growth tests of glucose fermentation. Anaerobic growth on glucose is impeded by the formate analog hypophosphite, an inhibitor of PflB [32], which is known to enter the cells via FocA [33]. We compared the anaerobic growth on glucose of cured UOF isolates G5876 (UOF1, *focA* F7C) and G5873 (UOF2, *focA* V97I) with their respective *focA* wild type derivatives G6759 (UOF1) and G6760 (UOF2) with or without added hypophosphite. Also, the *focA* wild type control strain G5416 (Δ*lpd*) and its derivatives carrying the *focA* mutations F7C (G6514) and V97I (G6515) were included in the experiment. As expected, the presence of hypophosphite lowered growth for all strains tested (Fig 5). No significant difference of hypophosphite impact between the non-evolved strains bearing either the wild type or a mutated *focA* allele was noted, suggesting an unaltered capacity of the mutated FocA variants to transport small anions into the cells. However, for the evolved strains, the presence of the mutated *focA* allele entailed a higher hypophosphite toxicity – and thus a higher influx of formate in the cells – with respect to the wild type, notably in case of allele F7C (Fig 5). As FocA is a bidirectional transporter, we also tested whether formate efflux was affected by the mutations by measuring the formate accumulation in the growth medium during fermentative proliferation under anaerobic conditions on glucose. As shown in Fig 6, formate secretion from the evolved strains G5873 and G5876 harboring *focA* mutation F7C and V97I, respectively, was comparable to the secretion by their derivatives G6759 and G6760, which harbor wild type *focA*. The control strain MG1655, the non-evolved Δ*lpd* strain G5416, and its derivatives G6514 (Δ*lpd focA* V97I) and G6515 (Δ*lpd focA* F7C), however, secreted around 70% more formate after 8 hours of growth. Presumably, continuous culture adaptation, independent of the FocA mutations, led to higher formate availability in the cells through lowered secretion. Together with the results from the hypophosphite inhibition experiment, a complex picture of the impact of the *focA* mutations selected during evolution under aerobic conditions is drawn. FocA is usually not or only marginally expressed in this condition, interactions between PflB or other cell components described for anaerobic growth could impact FocA mediated aerobic formate channeling in an unexplored way. A deeper analysis, involving structure-function assays, seems necessary to understand the mechanism of the transporter operating in the presence of oxygen and with a high external formate supply.

**Fig 7:**
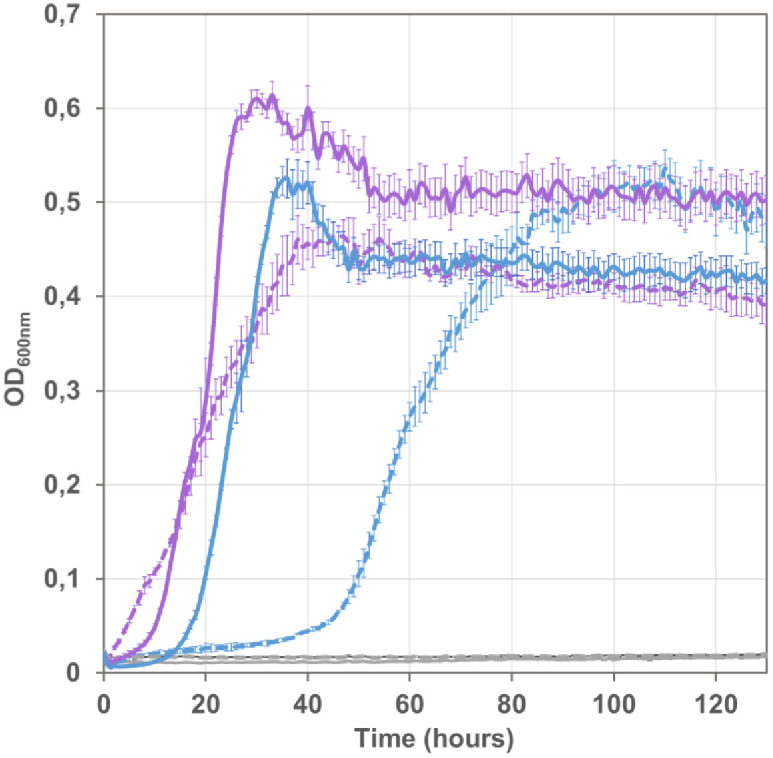
Influence of the genetic background on FDH-dependent growth. Formate-complemented growth of Δ*lpd* strains expressing *C. necator* or *Thiobacillus sp.* formate dehydrogenase was compared in unevolved genetic background of strain G5416 (broken line) and evolved genetic background of strain G5876 (cured derivative of UOF1 isolate G5823) (plain line). Growth of strains G6114 and G6217, derivatives of strain G5416 harboring plasmid pZE::CnFDH (blue broken line) or pZE::TsFDH (purple broken line), respectively, was compared with strains G6280 and G6272, derivatives of strain G5876 likewise harboring plasmids pZE::CnFDH (pGEN1393, blue line) or pZE::TsFDH (pGEN1395, purple line), respectively. Lack of growth of plasmid-free strains G5416 and G5876 (grey lines) demonstrate the dependence on heterologous FDH activity for cell proliferation under selective conditions. Bacteria were grown on mineral MS medium supplemented with formate (60 mM) acetate (20 mM) and pyruvate (20 mM) at 30°C in a Bioscreen C plate reader, shown are the mean values of three measurements and the standard deviation.

To test whether the growth rate enhancement effect of the UOF-background was somehow related to the activity of the *C. necator* FDH, we transformed the cured strain G5876 with a pZE21-derived plasmid (referred herein as pZE, see materials and methods) for constitutive expression containing the gene for formate dehydrogenase of *Thiobacillus* sp. KNK65MA (pGEN1395) [34] and compared its growth on formate as sole energy source with the unevolved strain G6272, also harboring the same plasmid. In contrast to the *C. necator* enzyme, the enzyme of *Thiobacillus* is monomeric not involving metal centers. Fig 7 shows that the evolved background, as was the case for the *C. necator* FDH, had an enhancing effect on growth under selective conditions when this enzyme was expressed.

**Fig 8:**
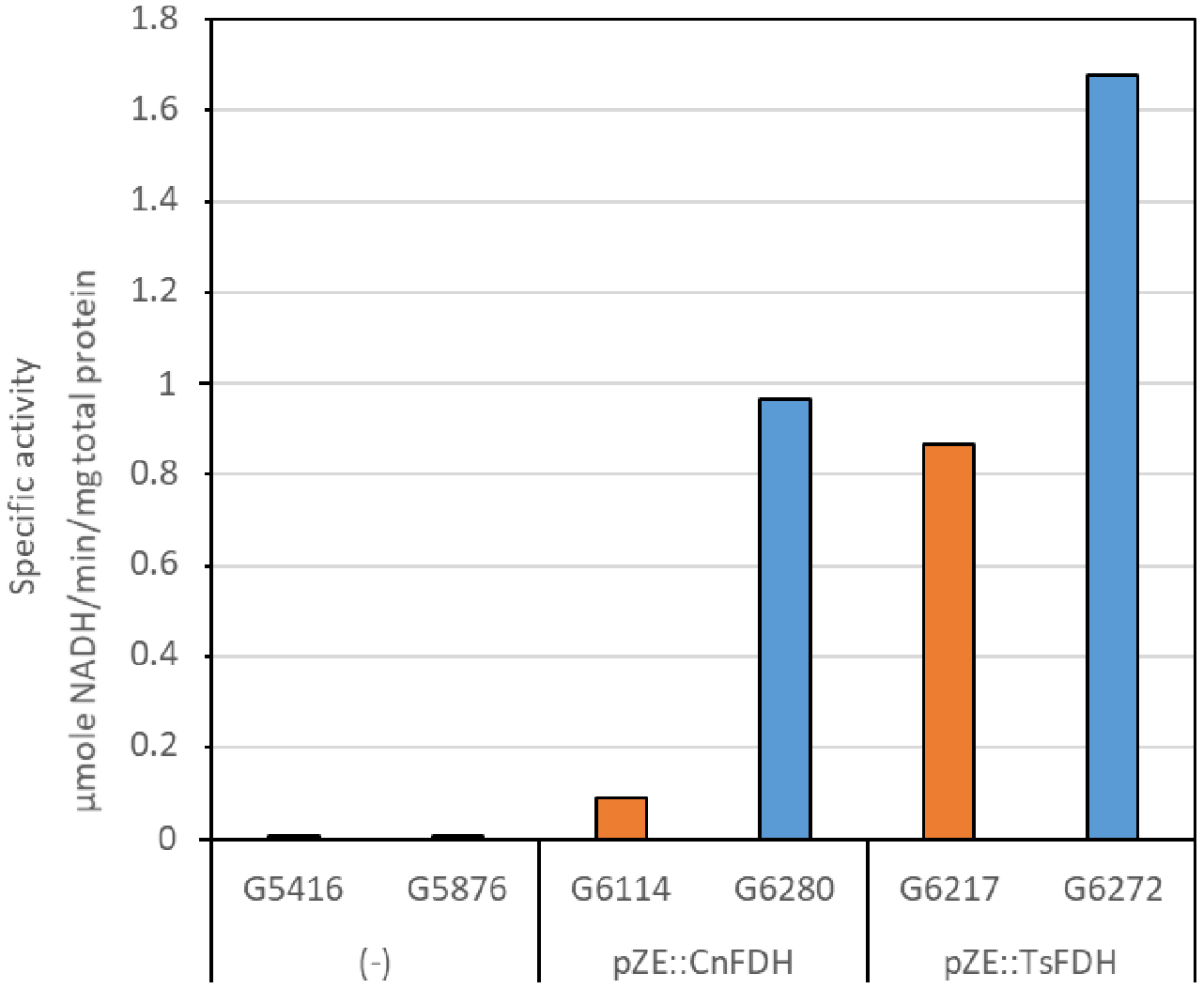
Formate oxidation by heterologous formate dehydrogenases in cell lysates obtained from an evolved Δ*lpd* strain (blue bars) is higher than obtained from a non-evolved Δ*lpd* strain background (orange bars). Cell lysates were prepared from control strains G5416 and G5876 grown in mineral MS medium supplemented with 0,2% glucose and acetate (20 mM) and from strains G6114, G6217, G6280 and G6272 grown in mineral MS medium supplemented with acetate (20 mM), pyruvate (20 mM) and formate (60 mM). Strains G6114 and G6217 were constructed from the MG1655 derived Δ*lpd* strain G5416 through transformation of plasmid pGEN1393 (pZE::CnFDH) and pGEN1395 (pZE::TsFDH), respectively (orange bars). Strains G6280 and G6272 were derived from the cured UOF1 isolate G5876 through transformation of plasmid pGEN1393 and pGEN1395, respectively (blue bars). Formate dehydrogenase specific activity of the lysates were determined.

We measured formate dehydrogenase activity in cell lysates of the four FDH expressing strains compared in Fig 7. A net increase in activity was observed for both enzymes when expressed from the evolved background, as compared with the non-evolved strains (Fig 8), with a greater gain for the FDH of *C. necator* (10-fold) than for the FDH of *Thiobacillus* (2-fold). Given that the FDH expressing pZE plasmids were not present in the cells during continuous culture adaptation, the activity gain seems to be due to enhanced expression and/or stability of the heterologous proteins. Moreover, lysates of the strains containing the FDH from *Thiobacillus* yielded higher specific activity than those containing the *C. necator* FDH. This higher activity might also reflect an incomplete maturation of CnFDH, as observed for metal-dependent heterologous enzymes expressed in *E. coli* [35]. No maturation is necessary for the *Thiobacillus* FDH. While these data are qualitatively in accordance with the growth measurements of Fig 7, the growth differences do not reflect the high enzyme activity variations. Possibly, other factors, like formate availability in the cells, play a role in determining the growth rates.

### Chromosomal integration of the complex FDH

**Fig 9:**
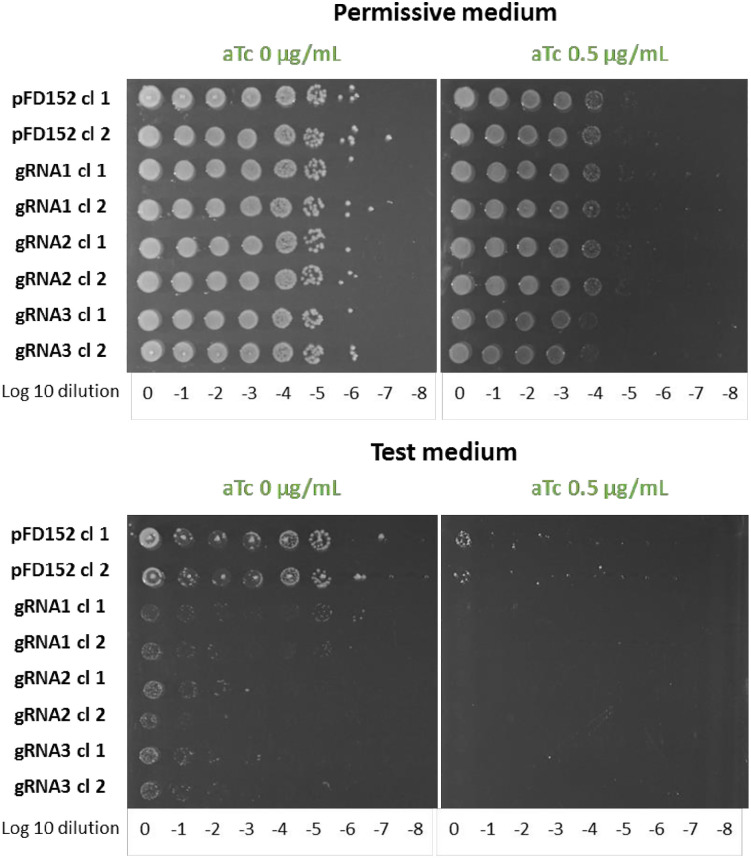
dCas9 silencing of *C. necator fdsGBACD* operon inserted in the chromosome of *E. coli* Δ*lpd* strain. Cells of strain G6435 (Δ*lpd* IS10::*fdsGBACD C.n.)* were grown overnight in permissive medium (MS glucose 0.2% acetate 20 mM), then serially diluted and dotted on semi-solid permissive or test medium (MS formate 60mM acetate 20mM pyruvate 20mM) containing or not the dCas9 inducer anhydrotetracycline (aTc) as indicated. Plates were incubated at 30°C for a maximum of seven days Results are shown for G6435 cells harboring the empty plasmid pFD152 as control, and G6435 cells expressing one of three different gRNAs cloned in pFD152.

To create a platform of stable *C. necator* FDH expression in *E. coli* enabling *in vivo* structure/function studies and the evolution of activity in continuous culture, we integrated the *fdsGBACD* operon in the IS10 insertion site of *E. coli* strain G5876 behind a strong promoter and an RBS following a described protocol [36,37] (see materials and methods for details). We used the evolved and plasmid-cured energy auxotrophic strain G5876 originating from culture UOF1 for chromosomal integration to favor our chances to obtain growth on formate. Formate-dependent growth was observed for the resulting strain G6435 and the essential implication of the integrated FDH operon demonstrated by CRISPR interference [38]. This method is based on the concomitant expression of a catalytically inactive dCas9 protein and a guide RNA targeting the gene or the operon to be silenced. The inactive dCas9 protein binds – guided by the gRNA - to the promoter or a gene locus near the N-terminus thus interfering with initiation or elongation of DNA transcription by the RNA polymerase. Fig 9 shows the results for the *C. necator fdsGBACD* operon silenced with three different gRNAs specific for the operon (see materials and methods). Overnight culture samples were serially diluted and dotted on permissive or test plates containing or not the dCas9 expression inducer anhydrotetracycline. Slight growth inhibition was noticed on permissive medium in the presence of the inducer, suggesting residual DNA cleavage activity of dCas9 independent of the presence of a gRNA. On test medium without inducer of dCas9 expression, growth of control cells was observed. Expression of a specific gRNA strongly impeded cell growth under these conditions, demonstrating loss of growth on formate through silencing of the formate dehydrogenase. In the presence of the inducer, virtually no growth was observed, reflecting the deleterious effect of residual dCas9 activity on the Δ*lpd* strains due to their attenuated growth on the test medium.

**Fig 10:**
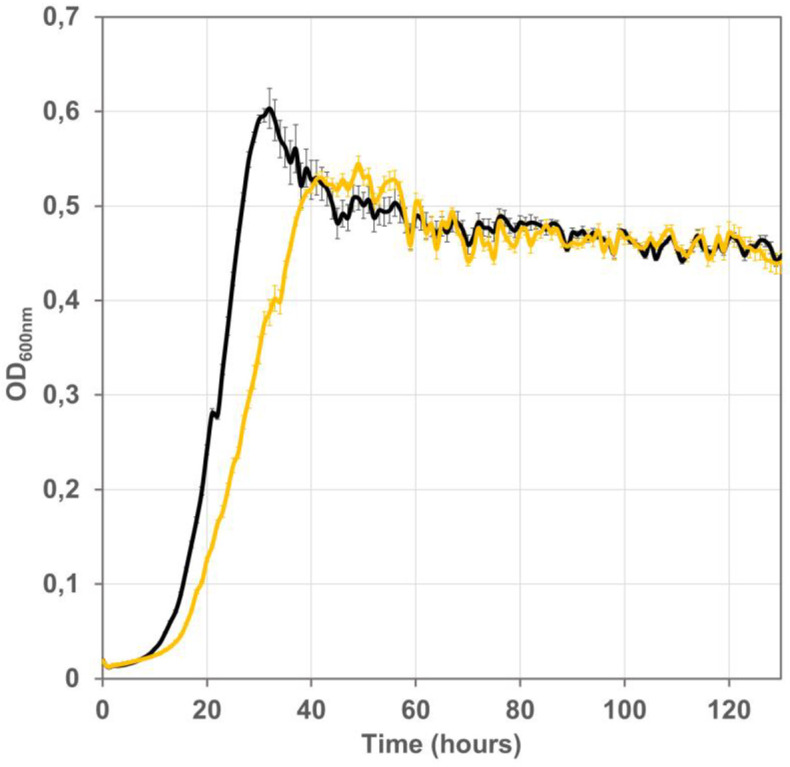
Impact of FDH expression context on growth. Growth of the UOF1 isolate G5823, which harbors plasmid pTRC-CnFDH (black line) was compared with growth of G5823 descendant strain G6435 containing of *C. necator* FDH operon *fdsGBACD* on the chromosome (yellow line). Bacteria were grown on mineral MS medium supplemented with formate (60 mM), acetate (20 mM) and pyruvate (20 mM) at 30°C in a Bioscreen C plate reader, shown are the mean values of three measurements and the standard deviation.

Strain G6435 (Table 1) grew with a generation time of 3h30 in the formate/acetate/pyruvate test medium. As expected, induced FDH expression from the multicopy-plasmid pTrc99a supported faster growth on formate (Tgen = 1h30) than expression from the single chromosomal copy integrated in strain G6435 (Fig 10).

We conducted quantitative PCR to measure the transcript levels of the *C. necator* FDH genes *fdsG*, *fdsA* and *fdsD* expressed in three different strains to evaluate the impact of the genetic background and the expression format (Table 2) on transcription of the CnFDH subunits. As expected, in strain G5663 (unevolved background, expression from pGEN1340) as well as in strains G5823 (evolved background, expression from pGEN1340) and G6435 (evolved background, expression from the chromosome), transcript levels for the three subunits declined with increasing distance from the promoter, reflecting lower processivity of the RNA polymerase towards the 3’ end of the polycistronic template [39]. Inter-strain comparisons revealed a higher transcript level for the three plasmid-borne *fds* genes expressed in the non-evolved cells as compared with the evolved cells. At first sight, this finding is in contradiction to comparative growth (Fig 7) and enzyme activity (Fig 8) results pointing to higher formate oxidation activity in the evolved background. Possibly, the adaptation leading to higher enzyme activity concerned mostly cofactor maturation and eventually the correct folding and higher oxygen tolerance of this metal-dependent protein, rather than transcription efficiency. The fact that the transcript level of the *fdsD* gene coding for a chaperone activity was higher in the chromosomal expression context could argue in this sense.

**Table 2:** Comparison of the transcriptional levels of the genes *fdsG, fdsA* and *fdsD* by quantitative PCR.

Strains	Genotype	Relative transcript levelΔCt^a^ Ct *fds gene C. necator* – Ct *panB E.coli*		
		*fdsG*	*fdsA*	*fdsD*
**G5663**	Δ*lpd* pGEN1340 (*fdsGBACD C.n.*)	−9.45 ± 0.32	−8.78 ± 0.07	−6.24 ± 0.09
**G5823**	Δ*lpd focA* F7C *infC* E95K pGEN1340 (*fdsGBACD C.n.*)	−8.77 ± 0.13	−8.13 ± 0.17	−4.33 ± 0.04
**G6435**	Δ*lpd focA* F7C *infC* E95K IS10::*fdsGBACD C.n.::kan*	−7.57 ± 0.19	−6.76 ± 0.28	−4.71 ± 0.06
**Inter-strain comparison**		ΔΔCt (*fold change* 2^-ΔΔCt^)^b^		
		*fdsG*	*fdsA*	*fdsD*
**G5823-G5663**		0.68 (*0.62*)	0.65 (*0.64*)	1.92 (*0.26)*
**G6435-G5823**		1.2 (*0.43*)	1.37 (*0.39*)	−0.48 (*1.39*)

a The relative levels of expression of *fds* genes were evaluated by the calculation of ΔCt (Ct Cycle threshold) using *E. coli panB* gene as internal control.

b Inter-strain differences of the level of expression of each *fds* gene were evaluated by the calculation of ΔΔCt.

## Conclusion

Complex metal- and NAD-dependent formate dehydrogenases have been identified and studied in recent years. Purification and *in vitro* activity tests under oxic conditions demonstrated oxygen resistance of these enzymes [6,7].

In this study we addressed the question whether such a dehydrogenase could stably function *in vivo* under aerobic conditions. An energy auxotrophic *E. coli* strain was used as test system to validate FDH activity for formate oxidation to generate NADH necessary for cell growth [21]. The soluble formate dehydrogenase from *C. necator* expressed from plasmids or from the chromosome supported stable formate-dependent growth in the presence of O_2_. The plasmid-bearing strain was evolved in continuous culture for faster growth for up to 400 generations, without loss of the selective formate/FDH dependency of the populations. Genomic sequencing revealed adaptive mutations in the genetic background of evolved isolates, while the sequence of the heterologous FDH operon remained unchanged. Mutations in the gene *focA* coding for a bidirectional formate transporter found in all isolates analyzed pointed to formate availability in the cells as limiting factor. The strong increase of FDH activity in cell lysates from evolved cells as compared to unevolved cells, also argues in favor of growth limitation due to FDH substrate shortage. The fact that non-synonymous mutations in the *focA* locus appeared during evolution and were fixed in both cultures is a clear sign of the significance of the transporter for formate dependent growth of the selection strain. This is further corroborated by the diminished growth rate observed for evolved strains harboring the wild type gene upon allelic exchange. Results from hypophosphite uptake and formate secretion assays conducted under anaerobic conditions demonstrated an intricate interplay between the impact of the mutations and the evolved strain background. The presence of a mutant *focA* allele slightly enhanced formate uptake in the evolved strains, but not in the non-evolved. By contrast, while no impact of the *focA* mutations on formate secretion was detectable, secretion was diminished for all evolved strains tested with respect to the non-evolved counterparts. The marked difference in formate secretion during anoxic growth between evolved and non-evolved strains, independent of the FocA version present, cannot easily be attributed to any of the other gene mutations on the grounds of the annotated activity of the respective gene product. Given that the strain evolution was conducted under aerobic conditions, the effect of the mutations on growth might be related to the presence of oxygen. While the results of the anaerobic assays point to a higher availability of formate in the evolved strains, they call for further experiments, among them intracellular dosage of formate and systematic reversions of the mutations, for a better understanding of the effects of the *focA* mutations.

The CnFDH chromosomal insertion construct is of special interest as it provides a stable expression platform not only for continuous culture evolution, but also for *in vivo* site directed or targeted random mutagenesis. In recent years, methods were developed to enable mutagenesis and selection in the same cellular background. Key residues directly involved in the catalytic activity could be identified giving insights into structure/function relationships of these complex enzymes by *in vivo* activity screens avoiding protein overexpression and purification.

As a further perspective, FDH activities could be enhanced for the reductive reaction, using recently constructed formate dependent *E. coli* strains as selection chassis [40,41]. Efficient enzymatic CO_2_ reduction to formate can be envisioned as an entry point for CO_2_ assimilation for biomass production engineered in initially heterotrophic model strains like *E. coli*. The metal- and NAD-dependent formate dehydrogenases, characterized by their oxygen tolerance and a CO_2_ reduction activity up to 20-fold higher as compared to non-metal FDHs, are promising candidates for the implementation of synthetic autotrophic growth modes.

## Materials and methods

### FDH plasmid constructions

The plasmids used and constructed in this study are listed in Table 1. The *fdsGBACD* operon (gene IDs 10917038–10917042) coding for the soluble Mo-dependent formate dehydrogenase of *Cupriavidus necator* N1 DSM 13513 (CnFDH) was PCR amplified from genomic DNA using oligonucleotide primers 6125 and 6126 (see table below for oligonucleotide primers used for cloning). Plasmid pTrc99a was PCR amplified using overlapping primers 6123 and 6124. Both amplification products were gel purified and used to assemble plasmid pGEN1340 employing the HiFi DNA Assembly Cloning Kit (New England Biolabs) for Gibson cloning. To assemble integrative plasmid pGEN1378, the *fdsGBACD* operon was PCR amplified from genomic *Cupriavidus necator* N1 DNA using oligonucleotide primers 6190 and 6191. Two sub-fragments of plasmid pKI_IS10 (gift of S. Wenk [36]) were PCR amplified using primer pairs 6212/6211 and 6256/6255, respectively. Together with the CnFDH amplification product, a tripartite assembly of gel purified PCR amplification products was conducted using the HiFi DNA Assembly Cloning Kit. Cloning was performed in *E. coli* DH5α λpir cells. CnFDH was also cloned into the pZE21-derived plasmid pFDH (gift from Ron Milo, Addgene 131706). Primers used for PCR amplification were 6316 and 6317 (pFDH backbone) and 6306 and 6307 (CnFDH) and the amplification products assembled following the CPEC protocol [42]. A version optimized for *E. coli* codon usage of the gene coding for formate dehydrogenase from *Thiobacillus sp*. (AB106890) was synthetized by Twist Biosciences, California and cloned into plasmid pFDH using the CPEC protocol [42]. Primers used for PCR amplification and CPEC assembly were 6304 and 6305 (pFDH backbone) and 6318 and 6319 (*Thiobacillus sp* FDH).

**Table:** 

pTrc99a	6123	GATGCTGGAGTAAAAGCTTGGCTGTTTTGGC
	6124	CAATTTCTGGCATTTAATTAACCTCGAATTCCATGG
pKI-IS10	6211	CAATTTCTGGCATCTCAGTACCTCCTCATTTTGTTTAAAGTTAAACAAAATTATTTCTATTA
	6212	GCGATGCTGGAGTAAGCTAGCGCGGCCGCCGCCGCAAA
	6255	CGATGACGTCACTGCCCGGCTGTAT
	6256	ATACAGCCGGGCAGTGACGTCATCG
pFDH	6304	ATGCTGGAGTAAGCTAGCGCGGCCG
	6305	CAATTTCTGGCATCTCAGTACCTCCTCATTTTGT
	6316	CTCAGTACCTCCTCATTTTGTTTAAAGTTAAACAAAA
	6317	TTAAGCTAGCGCGGCCG
*fdsGBACD*	6190	TAATAGAAATAATTTTGTTTAACTTTAAACAAAATGAGGAGGTACTGAGATGCCAGAAATTGCCCCCCACGCAGCG
	6191	TTTGCGGCGGCGGCCGCGCTAGCTTACTCCAGCATCGCCCGATGCCGCCCCA
	6125	GAGGTTAATTAAATGCCAGAAATTGCCCCCCAC
	6126	ACAGCCAAGCTTTTACTCCAGCATCGCCCGATG
	6306	GGAGGTACTGAGATGCCAGAAATTGCCCCCCA
	6307	CGGCCGCGCTAGCTTACTCCAGCATCGCCCGATGC
*Ts fdh*	6318	TTTTGTTTAACTTTAAACAAAATGAGGAGGTACTGAGATGGCAAAGATCTTATGTGTGTTATACGAC
	6319	CGGCCGCGCTAGCTTAATTAACCCGCTTTCTTAAATTTTGCCGC

### Strain constructions

The strains used or constructed in this study were all derivatives of the wild type *E. coli* K12 strain MG1655. Their relevant genotypes and filiations are listed in Table 1. The desired genetic contexts were obtained by phage P1-mediated transductions of gene knockouts substituted by antibiotic resistance cassettes according to the method of [43]. Genes of interest were mobilized in the desired recipient cells by co-transduction with closely linked kanamycin markers originating from the Keio *E. coli* knockout collection [44]. Resistance cassettes were removed by FLP-recombinase reaction after transformation with the plasmid pCP20. The *fdsGBACD* operon flanked in 3’ by a strong promoter derived from the *E. coli pgi*-promoter (pgi#20) [37] and an RBS was inserted in the chromosomal IS10 site of MG1655 by recombination with plasmid pKI_IS10_CnFDH [36]. The plasmid was transformed into the *E. coli pir*^+^ donor strain ST18 [45] and transferred into recipient strain MG1655 by conjugation. Plasmid integration and subsequent removal of the plasmid backbone were selected as described [36]. The *fdsGBACD* operon was PCR amplified and correct integration verified by sequencing.

### Continuous culture

Evolution experiments in continuous culture were carried out using GM3 fluidic self-cleaning cultivation devices. This device automatically dilutes growing cell suspensions with nutrient medium by keeping the culture volume constant. A continuous gas flow of controlled composition through the culture vessel ensures constant aeration and counteracts cell sedimentation. Twin culture vessels connected with silicone tubing enable the periodical transfer of the evolving culture between vessels and their cleaning upon rinsing with a 5N NaOH solution to remove biofilms [46].

To evolve G5663 cells to faster growth on formate as energy source, a turbidostat regime was programmed. This cultivation regime enables the selection of optimized growth in permissive conditions. Every 10 min, the optical density of the culture is automatically measured and compared to a fixed threshold (OD_600nm_ value of 0.4). When the measured OD_600nm_ exceeds the threshold, a pulse of fresh nutrient medium is injected into the culture and the same volume of used culture discarded. The dilutions ensure that the biomass in the vessel remains constant and that the bacteria grow at their maximal growth rate. A preculture of G5663 cells was grown in minimal saline medium MS supplemented with formate (60 mM) acetate (20 mM) pyruvate (20 mM) medium and IPTG (100 µM) at 30°C to an OD_600nm_ of 0,8 and used to inoculate two independent GM3 culture vessels (UOF1 and UOF2) with the same medium composition. Samples of the growing cultures were taken once a week and kept at −80°C. Growth was stopped after 230 (UOF1) and 380 (UOF2) generations and culture samples plated on semisolid MS formate acetate pyruvate medium to obtain isolates from colonies for further analysis.

### Bacterial growth assays

A Microbiology Reader Bioscreen C apparatus (Thermo Fisher Scientific) was used for growth curve recordings under aerobic conditions. It consists of a thermostatic incubator and a culture growth monitoring device (OD reader). Overnight bacterial cultures were washed once in MS medium and diluted 100-fold in the respective growth medium; 200 µl aliquots of the cell suspensions were distributed into honeycomb 100-wells plates. Each experiment was performed in triplicate. The plates were incubated at 30° or 37°C under continuous agitation. Bacterial growth was followed by recording optical densities at 600 nm every 15 minutes during the indicated time.

### Anaerobic assays

For tests under anaerobic conditions, cells were grown on glucose (20 g/L) in MM *E. coli* anaerobic medium [47] without nitrate and yeast extract in Wheaton serum glass bottles (Sigma). Tests to determine sensitivity of strains for the formate analog hypophosphite were conducted by growing the cells at 30°C under anaerobic conditions in MM medium in the presence or absence of 10 mM Na-hypophosphite.

The formate concentrations in samples from anaerobically grown cultures were determined by an enzymatic assay. The reaction took place in 100 µL of 0.1 M phosphate buffer, pH 7.4, containing 5 mM of NAD^+^ and 80 mU of the formate dehydrogenase from *Candida boidinii* (Sigma). After 2 hours of incubation at 30°C, the NADH absorbance was measured spectrophotometrically at 340_nm_ (Spectramax Plus, Molecular Device) and formate concentration calculated using a calibration curve obtained for the same conditions of reaction (formate concentration range: 0; 0.5; 1.5; 2 mM).

### Whole genome sequencing and mutation analysis

Pair-end libraries (2x150 bp) were prepared from 1 µg of genomic DNA of the evolved isolates and sequenced using a MiSeq sequencer (Illumina). High-throughput sequencing data were analyzed using the PALOMA bioinformatic pipeline implemented in the MicroScope platform [48] (https://mage.genoscope.cns.fr/microscope/home/). In a first step, reads were mapped onto the *E. coli* MG1655 reference (NC_000913.3) using the SSAHA2 package (v.2.5.1). Only unique matches having an alignment score equal to at least half of their length were retained as seeds for full Smith-Waterman realignment [49] with a region extended on both sides by five nucleotides of the reference genome. All computed alignments then were screened for discrepancies between read and reference sequences and a score based on coverage, allele frequency, quality of bases, and strand bias was computed for each detected event to assess its relevance. The mutations (single nucleotide variations and short insertions or deletions) with a score superior to 0.8 with at least five supporting reads were retained.

### Gene silencing

Gene silencing was performed using CRISPRi method [38]. Specific gRNAs were cloned into plasmid pFD152 (gift from Solange Miele) harboring the inducible gene coding for dCas9 and a gRNA cloning sites. Three gRNAs (gRNA1: GGCGCCACGTGGTACAGGTC, gRNA2: AGGTGCATGGCGTGATCACC, gRNA3: AAGCGCTGGCCGAGCATGCG) were cloned into plasmid pFD152 using Golden Gate technique (Bsa I) and tested for silencing of the *fdsGBACD* operon. Plasmids expressing a specific gRNA were transformed into the strain G6435 and serial dilutions of overnight cultures were dotted on large Petri dishes in permissive and test conditions with and without induction of dCas9 by 0.5 µg/ml anhydrotetracycline and incubated for 2–7 days at 30°C.

### Expression analysis by reverse transcriptase quantitative PCR (RT-qPCR)

The mRNA levels of *fdsA, fdsG* and *fdsD* genes were determined by RT-qPCR with *panB* as internal standard for expression normalization. Cultures were perfomed in MS mineral medium supplemented with acetate 20 mM, pyruvate 20 mM, formate 60 mM and IPTG 0.1 mM if necessary. Cells were harvested in exponential phase (OD_600nm_ 0.5–0.6). Total RNA was extracted using the RNeasy Mini Kit (Qiagen, Hilden, Germany) following the manufacturer’s instructions. In summary, 2 volumes of RNAprotect Bacteria Reagent (Qiagen, Hilden, Germany) are added to one volume of bacterial culture. After pelleting the cells, RNA extraction was performed and RNA treated with DNase I (NEB). The quality of the extraction was verified by agarose gel electrophoresis. cDNA were generated through reverse transcription using High capacity cDNA Reverse transcription kit (Applied Biosystems) and their concentrations were determined using Qubit™ ssDNA Assay Kit (Invitrogen). Quantitative real-time PCR experiments were performed for two technical replicates, each analyzed for four template dilutions using the KAPA SYBR FAST kit (Roche). Primer pairs used for amplification of genes *fdsA, fdsG* and *fdsD* and *panB* are listed in the table below:

**Table:** 

*panB*	4463	TTAGAAGCTGCTGGGGCACA
	4464	CCGTTTCGGCGAGGAAATT
*fdsG*	6419	ATCCTGCATGAGATCCAGGACAC
	6420	AAGTGGTGGTAGAAGGTGATCACG
*fdsA*	6422	AACGGCAATTGCGAACTGCAG
	6423	ATTCGTCCTTCTTCATCTGCGTGTG
*fdsD*	6427	ACAACCTCATCACCATGGCCAAC
	6428	ATATCCAACAGCCCGTTCCCTG

### Determination of formate dehydrogenase specific activity in cell lysates

Small volume precultures of Δ*lpd* strains expressing or not a heterologous formate dehydrogenase from a pZE-plasmid were prepared in permissive MS glucose 0.2% formate (60 mM) acetate (20 mM) pyruvate (20 mM) medium supplemented with spectinomycin when needed (100 mg/L) at 30°C and used to inoculate either 500 ml of selective MS formate (60 mM) acetate (20 mM) pyruvate (20 mM) medium or 500 mL permissive medium (control strains without plasmid). Cultures grew to an OD_600nm_ between 0.8 and 1.0. They were centrifuged and the cell pellet suspended in 5 ml of lysis buffer (100 mM TRIS pH 7.0, 10 mM KNO_3_, 1 mM dithiothreitol, 0.1 mM pefablock and 10% glycerol) and sonicated using an Ultrasonic processor. After centrifugation, total protein content of the clarified extract was measured using the Bradford method (Bio-Rad protein assay dye) in the clarified cell extract, and formate dehydrogenase specific activity determined. Activity assays were performed in 120 µL of activity buffer (100 mM TRIS pH 9.0, 100 mM formate) containing 5 mM of NAD^+^ and the reaction was initiated by addition of various volumes of cell lysate. Variation of absorbance was recorded spectrophotometrically at 340_nm_ (Safas UV mc2 double beam spectrophotometer) and initial rate of NADH production was determined using a molar extinction coefficient of 6220 M^-1^ cm^-1^ and residual NADH formation in the absence of formate subtracted.

## Supporting information

S1 File Mutations fixed in the evolved strains. (DOCX)

S2 File Experimental raw data. (XLSX)

## References

[1] Ferry JG. Formate dehydrogenase. FEMS Microbiol Rev. 1990;7(3–4):377–82. doi: 10.1111/j.1574-6968.1990.tb04940.x 2094290

[2] Popov VO, Lamzin VS. NAD(+)-dependent formate dehydrogenase. Biochem J. 1994;301 ( Pt 3)(Pt 3):625–43. doi: 10.1042/bj3010625 8053888 PMC1137035

[3] Zhang L, Liu J, Ong J, Li SFY. Specific and sustainable bioelectro-reduction of carbon dioxide to formate on a novel enzymatic cathode. Chemosphere. 2016;162:228–34. doi: 10.1016/j.chemosphere.2016.07.102 27501309

[4] Son EJ, Ko JW, Kuk SK, Choe H, Lee S, Kim JH, et al. Sunlight-assisted, biocatalytic formate synthesis from CO2 and water using silicon-based photoelectrochemical cells. Chem Commun (Camb). 2016;52(62):9723–6. doi: 10.1039/c6cc04661d 27411734

[5] Maia LB, Moura I, Moura JJG. Molybdenum and tungsten-containing formate dehydrogenases: aiming to inspire a catalyst for carbon dioxide utilization. Inorganica Chimica Acta. 2017;455:350–63. doi: 10.1016/j.ica.2016.07.010

[6] Friedebold J, Bowien B. Physiological and biochemical characterization of the soluble formate dehydrogenase, a molybdoenzyme from Alcaligenes eutrophus. J Bacteriol. 1993;175(15):4719–28. doi: 10.1128/jb.175.15.4719-4728.1993 8335630 PMC204923

[7] Yu X, Niks D, Mulchandani A, Hille R. Efficient reduction of CO2 by the molybdenum-containing formate dehydrogenase from Cupriavidus necator (Ralstonia eutropha). J Biol Chem. 2017;292(41):16872–9. doi: 10.1074/jbc.M117.785576 28784661 PMC5641872

[8] Yu X, Niks D, Ge X, Liu H, Hille R, Mulchandani A. Synthesis of formate from CO2 gas catalyzed by an O2-tolerant NAD-dependent formate dehydrogenase and glucose dehydrogenase. Biochemistry. 2019;58(14):1861–8. doi: 10.1021/acs.biochem.8b01301 30839197

[9] Radon C, Mittelstädt G, Duffus BR, Bürger J, Hartmann T, Mielke T, et al. Cryo-EM structures reveal intricate Fe-S cluster arrangement and charging in Rhodobacter capsulatus formate dehydrogenase. Nat Commun. 2020;11(1):1912. doi: 10.1038/s41467-020-15614-0 32313256 PMC7171172

[10] Amao Y. Formate dehydrogenase for CO2 utilization and its application. J CO2 Util. 2018;26:623–41. doi: 10.1016/j.jcou.2018.06.022

[11] Ma Z, Legrand U, Pahija E, Tavares JR, Boffito DC. From CO2 to formic acid fuel cells. Ind Eng Chem Res. 2020;60(2):803–15. doi: 10.1021/acs.iecr.0c04711

[12] Yishai O, Lindner SN, Gonzalez de la Cruz J, Tenenboim H, Bar-Even A. The formate bio-economy. Curr Opin Chem Biol. 2016;35:1–9. doi: 10.1016/j.cbpa.2016.07.005 27459678

[13] Yishai O, Goldbach L, Tenenboim H, Lindner SN, Bar-Even A. Engineered assimilation of exogenous and endogenous formate in *Escherichia coli*. ACS Synth Biol. 2017;6(9):1722–31. doi: 10.1021/acssynbio.7b00086 28558223

[14] Nielsen CF, Lange L, Meyer AS. Classification and enzyme kinetics of formate dehydrogenases for biomanufacturing via CO2 utilization. Biotechnol Adv. 2019;37(7):107408. doi: 10.1016/j.biotechadv.2019.06.007 31200015

[15] Schuchmann K, Müller V. Direct and reversible hydrogenation of CO2 to formate by a bacterial carbon dioxide reductase. Science. 2013;342(6164):1382–5. doi: 10.1126/science.1244758 24337298

[16] Çakar MM, Ruupunen J, Mangas-Sanchez J, Birmingham WR, Yildirim D, Turunen O, et al. Engineered formate dehydrogenase from Chaetomium thermophilum, a promising enzymatic solution for biotechnical CO2 fixation. Biotechnol Lett. 2020;42(11):2251–62. doi: 10.1007/s10529-020-02937-7 32557118

[17] Bruinsma L, Wenk S, Claassens NJ, Martins Dos Santos VAP. Paving the way for synthetic C1 - Metabolism in Pseudomonas putida through the reductive glycine pathway. Metab Eng. 2023;76:215–24. doi: 10.1016/j.ymben.2023.02.004 36804222

[18] Sawers G. The hydrogenases and formate dehydrogenases of Escherichia coli. Antonie Van Leeuwenhoek. 1994;66(1–3):57–88. doi: 10.1007/BF00871633 7747941

[19] Oh JI, Bowien B. Structural analysis of the fds operon encoding the NAD+-linked formate dehydrogenase of Ralstonia eutropha. J Biol Chem. 1998;273(41):26349–60. doi: 10.1074/jbc.273.41.26349 9756865

[20] Hille R, Hall J, Basu P. The mononuclear molybdenum enzymes. Chem Rev. 2014;114(7):3963–4038. doi: 10.1021/cr400443z 24467397 PMC4080432

[21] Wenk S, Schann K, He H, Rainaldi V, Kim S, Lindner SN, et al. An “energy-auxotroph” Escherichia coli provides an in vivo platform for assessing NADH regeneration systems. Biotechnol Bioeng. 2020;117(11):3422–34. doi: 10.1002/bit.27490 32658302

[22] Gennis RB, Hager LP. Pyruvate oxidase. In: The enzymes of bioligical membranes. Springer US; 1976. p. 493–504. doi: 10.1007/978-1-4684-2655-7_14

[23] Abdel-Hamid AM, Attwood MM, Guest JR. Pyruvate oxidase contributes to the aerobic growth efficiency of Escherichia coli. Microbiology (Reading). 2001;147(Pt 6):1483–98. doi: 10.1099/00221287-147-6-1483 11390679

[24] Kammel M, Pinske C, Sawers RG. FocA and its central role in fine-tuning pH homeostasis of enterobacterial formate metabolism. Microbiology (Reading). 2022;168(10):10.1099/mic.0.001253. doi: 10.1099/mic.0.001253 36197793

[25] Peters K, Sargent F. Formate hydrogenlyase, formic acid translocation and hydrogen production: dynamic membrane biology during fermentation. Biochim Biophys Acta Bioenerg. 2023;1864(1):148919. doi: 10.1016/j.bbabio.2022.148919 36152681

[26] Clark DP. The fermentation pathways of Escherichia coli. FEMS Microbiol Rev. 1989;5(3):223–34. doi: 10.1016/0168-6445(89)90033-8 2698228

[27] Sawers G, Watson G. A glycyl radical solution: oxygen-dependent interconversion of pyruvate formate-lyase. Mol Microbiol. 1998;29(4):945–54. doi: 10.1046/j.1365-2958.1998.00941.x 9767563

[28] Kammel M, Hunger D, Sawers RG. The soluble cytoplasmic N-terminal domain of the FocA channel gates bidirectional formate translocation. Mol Microbiol. 2021;115(4):758–73. doi: 10.1111/mmi.14641 33169422

[29] Rossmann R, Sawers G, Böck A. Mechanism of regulation of the formate-hydrogenlyase pathway by oxygen, nitrate, and pH: definition of the formate regulon. Mol Microbiol. 1991;5(11):2807–14. doi: 10.1111/j.1365-2958.1991.tb01989.x 1779767

[30] Sawers G, Böck A. Novel transcriptional control of the pyruvate formate-lyase gene: upstream regulatory sequences and multiple promoters regulate anaerobic expression. J Bacteriol. 1989;171(5):2485–98. doi: 10.1128/jb.171.5.2485-2498.1989 2651404 PMC209925

[31] Hopper S, Böck A. Effector-mediated stimulation of ATPase activity by the sigma 54-dependent transcriptional activator FHLA from *Escherichia coli*. J Bacteriol. 1995;177(10):2798–803. doi: 10.1128/jb.177.10.2798-2803.1995 7751289 PMC176951

[32] Plaga W, Frank R, Knappe J. Catalytic-site mapping of pyruvate formate lyase. Hypophosphite reaction on the acetyl-enzyme intermediate affords carbon-phosphorus bond synthesis (1-hydroxyethylphosphonate). Eur J Biochem. 1988;178(2):445–50. doi: 10.1111/j.1432-1033.1988.tb14468.x 3061816

[33] Suppmann B, Sawers G. Isolation and characterization of hypophosphite--resistant mutants of Escherichia coli: identification of the FocA protein, encoded by the pfl operon, as a putative formate transporter. Mol Microbiol. 1994;11(5):965–82. doi: 10.1111/j.1365-2958.1994.tb00375.x 8022272

[34] Nanba H, Takaoka Y, Hasegawa J. Purification and characterization of an alpha-haloketone-resistant formate dehydrogenase from Thiobacillus sp. strain KNK65MA, and cloning of the gene. Biosci Biotechnol Biochem. 2003;67(10):2145–53. doi: 10.1271/bbb.67.2145 14586102

[35] Hartmann T, Leimkühler S. The oxygen-tolerant and NAD -dependent formate dehydrogenase from Rhodobacter capsulatus is able to catalyse the reduction od CO2 to formate. 2013. https://doi.org/10/1111/febs.12528 24034888 10.1111/febs.12528 24034888

[36] Wenk S, Yishai O, Lindner SN, Bar-Even A. An engineering approach for rewiring microbial metabolism. Methods Enzymol. 2018;608:329–67. doi: 10.1016/bs.mie.2018.04.026 30173769

[37] Braatsch S, Helmark S, Kranz H, Koebmann B, Jensen PR. Escherichia coli strains with promoter libraries constructed by Red/ET recombination pave the way for transcriptional fine-tuning. Biotechniques. 2008;45(3):335–7. doi: 10.2144/000112907 18778259

[38] Depardieu F, Bikard D. Gene silencing with CRISPRi in bacteria and optimization of dCas9 expression levels. Methods. 2020;172:61–75. doi: 10.1016/j.ymeth.2019.07.024 31377338

[39] Wang Y, Yue X-J, Yuan S-F, Hong Y, Hu W-F, Li Y-Z. Internal promoters and their effects on the transcription of operon genes for epothilone production in Myxococcus xanthus. Front Bioeng Biotechnol. 2021;9:758561. doi: 10.3389/fbioe.2021.758561 34778232 PMC8579030

[40] Kim S, Lindner SN, Aslan S, Yishai O, Wenk S, Schann K, et al. Growth of E. coli on formate and methanol via the reductive glycine pathway. Nat Chem Biol. 2020;16(5):538–45. doi: 10.1038/s41589-020-0473-5 32042198

[41] Delmas VA, Perchat N, Monet O, Fouré M, Darii E, Roche D, et al. Genetic and biocatalytic basis of formate dependent growth of Escherichia coli strains evolved in continuous culture. Metab Eng. 2022;72:200–14. doi: 10.1016/j.ymben.2022.03.010 35341982

[42] Quan J, Tian J. Circular polymerase extension cloning for high-throughput cloning of complex and combinatorial DNA libraries. Nat Protoc. 2011;6(2):242–51. doi: 10.1038/nprot.2010.181 21293463

[43] Miller JH. Experiments in molecular genetics. Cold Spring Harbor, NY: Cold Spring Harbor Laboratory Press; 1972.

[44] Baba T, Ara T, Hasegawa M, Takai Y, Okumura Y, Baba M, et al. Construction of Escherichia coli K-12 in-frame, single-gene knockout mutants: the Keio collection. Mol Syst Biol. 2006;2:2006.0008. doi: 10.1038/msb4100050 16738554 PMC1681482

[45] Jackson SA, Fellows BJ, Fineran PC. Complete genome sequences of the Escherichia coli donor strains ST18 and MFDpir. Microbiol Resour Announc. 2020;9(45):e01014-20. doi: 10.1128/MRA.01014-20 33154010 PMC7645665

[46] Mutzel R, Marliere P. Method and device for selecting accelerated proliferation of living cells in suspension. Patent WO2000034433 A1. 2000. https://doi.org/WO2000034433A1

[47] Meynial-Salles I, Soucaille P. Creation of new metabolic pathways or improvement of existing metabolic enzymes by *in vivo* evolution in *Escherichia coli*. Methods Mol Biol. 2012;834:75–86. doi: 10.1007/978-1-61779-483-4_6 22144354

[48] Vallenet D, Calteau A, Dubois M, Amours P, Bazin A, Beuvin M, et al. MicroScope: an integrated platform for the annotation and exploration of microbial gene functions through genomic, pangenomic and metabolic comparative analysis. Nucleic Acids Res. 2020;48(D1):D579–89. doi: 10.1093/nar/gkz926 31647104 PMC7145621

[49] Smith TF, Waterman MS. Identification of common molecular subsequences. J Mol Biol. 1981;147(1):195–7. doi: 10.1016/0022-2836(81)90087-5 7265238

