# Supplementary material for: Functional expression of a Mo-dependent formate dehydrogenase in Escherichia coli under aerobic conditions
Source: PLoS One. 2025 Oct 29;20(10):e0334613. doi: 10.1371/journal.pone.0334613 (PMC12571268; doi:10.1371/journal.pone.0334613)
Supplement: S1 File — (DOCX) [file pone.0334613.s001.docx]

**Supplementary Table 1. Mutations fixed in the genome of the evolved strains**

| **UOF1** | chromosomal position* | mutation type | mutation event | *mutated gene* | intragenic position | amino acid change | intergenic mutation (distance to the flanking genes) | encoded activity | mutations identified in the indicated strain (•) | | |
| --- | --- | --- | --- | --- | --- | --- | --- | --- | --- | --- | --- |
|  |  |  |  |  |  |  |  |  | **G5823** | **G5824** | **G5825** |
|  | 953670 | A/C | SNP | *focA* | 20 | F7C |  | formate channel | • | • | • |
|  | 1029182 | C/A | SNP |  |  |  | yccW/yccX (-77/-105) |  | • | • | • |
|  | 1798380 | C/T | SNP | *infC* | 283 | E95K |  | translation initiation factor IF-3 | • | • | • |
|  |  |  |  |  |  |  |  |  |  |  |  |
| **UOF2** | chromosomal position* | mutation type | mutation event | *mutated gene* | intragenic position | amino acid change | intergenic mutation (distance to the flanking genes) | encoded activity | mutations identified in the indicated strain (•) | | |
|  |  |  |  |  |  |  |  |  | **G5848** | **G5849** | **G5850** |
|  | 88110 | A/G | SNP | *cra* | 83 | Y28C |  | DNA-binding transcriptional dual regulator |  |  | • |
|  | 953401 | C/T | SNP | *focA* | 289 | V97I |  | formate channel | • | • |  |
|  | 953670 | A/C | SNP | *focA* | 20 | F7C |  | « |  |  | • |
|  | 1785132 | G/A | SNP | *pps* | 5 | S2F |  | phosphoenolpyruvate synthetase | • | • |  |
|  | 2533860 | G/T | SNP | *crr* | 5 | G2V |  | enzyme IIA^Glc^ | • | • |  |
|  | 4159247 | T/G | SNP | *fabR* | 158 | L53W |  | DNA-binding transcriptional repressor of type II fatty acid synthase enzymes | • | • |  |
